# Survey of WU and KI polyomaviruses, coronaviruses, respiratory syncytial virus and parechovirus in children under 5 years of age in Tehran, Iran

**Published:** 2020-04

**Authors:** Fahimeh Sadat Aghamirmohammadali, Kaveh Sadeghi, Nazanin Zahra Shafiei-Jandaghi, Zahra Khoban, Talat Mokhtari-Azad, Jila Yavarian

**Affiliations:** Department of Virology, School of Public Health, Tehran University of Medical Sciences, Tehran, Iran

**Keywords:** Polyomaviruses, Human coronaviruses, Human respiratory syncytial virus, Human parechovirus, Children, Respiratory infections

## Abstract

**Background and Objectives:**

Severe acute respiratory infections (SARI) remain an important cause for childhood morbidity worldwide. We designed a research with the objective of finding the frequency of respiratory viruses, particularly WU and KI polyomaviruses (WUPyV & KIPyV), human coronaviruses (HCoVs), human respiratory syncytial virus (HRSV) and human parechovirus (HPeV) in hospitalized children who were influenza negative.

**Materials and Methods:**

Throat swabs were collected from children younger than 5 years who have been hospitalized for SARI and screened for WUPyV, KIPyV, HCoVs, HRSV and HPeV using Real time PCR.

**Results:**

A viral pathogen was identified in 23 (11.16%) of 206 hospitalized children with SARI. The rate of virus detection was considerably greater in infants <12 months (78.2%) than in older children (21.8%). The most frequently detected viruses were HCoVs with 7.76% of positive cases followed by KIPyV (2%) and WUPyV (1.5%). No HPeV and HRSV were detected in this study.

**Conclusion:**

This research shown respiratory viruses as causes of childhood acute respiratory infections, while as most of mentioned viruses usually causes mild respiratory diseases, their frequency might be higher in outpatient children. Meanwhile as HRSV is really sensitive to inactivation due to environmental situations and its genome maybe degraded, then for future studies, we need to use fresh samples for HRSV detection. These findings addressed a need for more studies on viral respiratory tract infections to help public health.

## INTRODUCTION

Acute respiratory tract infections (ARTI) affect children with high morbidity and mortality annually (1). The common respiratory viruses include influenza A and B (IFV), parainfluenza viruses (PIV) 1–4, human respiratory syncytial virus (HRSV), rhinoviruses (RV), adenoviruses (ADV), metapneumoviruses, bocaviruses and human coronaviruses (HCoV). These viruses are well recognized particularly among infants and preschool children (2).

Seven HCoVs have been recognized to date, HCoV 229E, OC43, HKU1 and NL63 are known to continuously circulate in the human population, particularly in young children (3). Infections with these HCoVs can lead to hospitalization of young kids and of old and immunocompromised patients (4). The other three viruses in this family are severe acute respiratory syndrome coronavirus (SARS-CoV), Middle East respiratory syndrome coronavirus (MERS-CoV) and SARS-CoV2 which are highly pathogenic (5). HCoV 229E and NL63 are classified in alphacoronavirus genus and the other HCoVs in betacoronavirus genus of family *Coronaviridae*.

Human respiratory syncytial virus is an important pathogen in children with ARTI and every year many young children infected with HRSV die, especially in developing countries (6). HRSV is classified in orthopneumovirus genus of *Pneumoviridae* family.

Two novel emerged viruses, KI (Karolinska Institute) (7) and WU (Washington University) (8) classified in betapolyomavirus genus and *Polyomaviridae* family. These viruses were detected in respiratory tract samples among children for the first time in 2007 (9). Human polyomavirus infections are usually asymptomatic and occur during childhood or early adolescence, resulting in primary viral replication and virus persistence close to the point of entry site, however across the lifespan the site of persistent infection in KI and WU polyomaviruses (KIPyV, WUPyV) remains unclear.

WU and KI viruses have been identified to utilize respiratory route of transmission. The primary viral replication initiates upon entry in tonsils and respiratory tract and this replication is often without clinical symptoms, but in immunocompromised children, they can cause symptomatic infections (10).

Human parechovirus (HPeV) is a genus of *Picornaviridae* family that has newly been identified as a cause of acute respiratory tract infections (RTI). This virus was detected during a summer outbreak of diarrhea in 1956 in the USA and described as echovirus 22 and 23 in the enterovirus (EV) genus of *Picornaviridae* family. HPeVs can cause a wide range of infections from subclinical to mild respiratory infection, gastroenteritis, sepsis, hepatitis and central nervous system infections particularly in young children (11).

Based on the epidemiological studies, HCoVs, HRSV, HPeVs, KIPyV and WUPyV have different prevalence in diverse regions. In Iran, there are some studies showing the prevalence of HCoVs and HRSV but there is a paucity of data indicating the prevalence of KIPyV, WUPyV and HPeV viruses in children less than 5 years of age with respiratory infections in Iran.

The aim of this study was to determine the prevalence of HCoVs, HRSV, HPeVs, KIPyV and WUPyV in respiratory samples collected from children under 5 years of age with SARI in order to improve surveillance approaches in prevention and treatment of respiratory diseases.

## MATERIALS AND METHODS

### Specimen collection.

This cross sectional study was performed on influenza negative throat swab samples collected from children under 5 years of age with severe acute respiratory infections (SARI) between 2017 and 2018 in National Influenza Center, Virology Department, School of Public Health, Tehran University of Medical Sciences, Iran. SARI is defined as an acute respiratory illness of recent onset (within seven days) manifested by fever (≥38 °C), cough and shortness of breath or difficulty in breathing requiring hospitalization.

### Nucleic acid extraction & cDNA synthesis.

Nucleic Acid was extracted from 200 μl of samples using the High Pure Viral Nucleic acid extraction kit (Roche Life Science) according to the manufacturer’s instruction for both viral RNA (HPeV, HRSV, HCoVs) and DNA (KIPyV, WUPyV). cDNA synthesis for RNA genome was carried out in 20 μl reaction mixture containing 4 μl of 5× reaction buffer, 2 μl of mixed dNTPs, 1 μl of RT-MULV enzyme (Fermentas, Germany), 2 μl random hexamer (Fermentas), 7 μl RNA template and 4 μl RNase/DNase free water. The mixture was incubated at 25 °C for 10 min, 42 °C for 60 min and 70 °C for 10 min.

### PCR reactions, One step Real time PCR: HPeV detection.

Taq-Man Real time PCR method with PrimerDesign ^TM^ Ltd (Primer Design, United Kingdom) kit was used to detect HPeV according to the manufacturer’s instruction. The 20 μl PCR reaction was consisted of 10 μl Oasing lyophilized onestep qRT-PCR mastermix, 0.5 μl of HPeV primer/probe mix, 2 μl of RNase/DNase free water and 7.5 μl of template. The amplification process was performed in Step One Plus Real time PCR system instrument (ABI, USA), according to the cycling protocol: 55 °C for 10 min (reverse transcription) and 95 °C for 2 min (DNA polymerase activation), followed by 50 cycles of 95 °C for 10s (denaturation) and 60 °C for 60s (annealing and extension).

### KIPyV and WUPyV detection: multiplex real time PCR.

Multiplex Real time PCR method, targeting KIPyV and WUPyV, was implemented with the specific primers and probes sets (12). Briefly, 5 μl of the nucleic acid extraction was added to 20 μl of reaction mixture containing 12.5 μl of 2× reaction buffer (Ampliqon master mix), 1.5 μl of WU primers-probe (10 pM), 1.5 μl of KI primers-probe (10 pM) and 4.5 μl of RNase/DNase free water. Temperature and time profiles were as follows: 95 °C for 5 min then 40 cycle of 95 °C for 15s, 55 °C for 20s, and 72 °C for 20s. In this study synthetic genome of KIPyV and WUPyV was used as positive controls and ddH _2_ O as negative control.

### HCoVs and HRSV detection: Real time PCR.

Real time PCR targeting nucleoprotein gene for detection of four HCoVs was performed as described previously (13). For HRSV detection, in house primers and probe targeting nucleoprotein gene were used. SenseN6: GCT AAA AGA AAT GGG AGA GG, antisN6: TAA TCA CGG CTG TAA GAC CA and probeN6: FAM-AGC TCC AGA ATA CAG GCA TGA C-BHQ1. For PCR reaction, 5 μl of cDNA was added to 20 μl of reaction mixture containing 12.5 μl of 2× reaction buffer (Ampliqon master mix), 1.5 μl of primers-probe (10 pM), and 3 μl of RNase/DNase free water. Temperature and time profiles were as follows: 95 °C for 5 min then 40 cycles of 95 °C for 15s, 54 °C for 30s and 60 °C for 30s.

## RESULTS

### Detection of HPeV, HRSV, KIPyV, WUPyV and HCoVs.

In throat swab samples collected from 206 children under 5 years of age, totally 23 cases (11/16%) were positive which, 3 (1.5%) were WUPyV, 4 (2%) were KIPyV and 16 (7.76%) were HCoVs. There were two cases of co-infections, one had 229E/HKU1 and the other case had NL63/HKU1. Among HCoVs, there were 7 (3.40%) 229E, 5 (2.42%) HKU1, 3 (1.45%) OC43 and 1 (0.49%) NL63. Further, no HPeV and HRSV could be detected in current study.

### Demographic characteristics.

In this study there were 117 (57%) males and 89 (43%) females with a sex ratio of 1.3:1. Of the 23 (11.29%) detected respiratory viral positive cases, 10 (4.85%) were females and 13 (6.31%) were males.

**Fig. 1.**
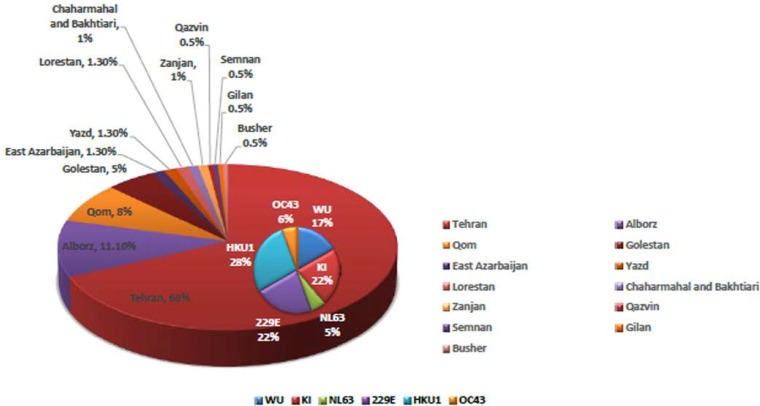
Distribution of children with SARI and positive samples according to the geographical region.

As shown in Table 1, the children with SARI were more frequently under 1 year of age. As shown in Fig. 1, Tehran province had the most positive cases as it had the most samples. Also, 229E virus in Qom, Golestan and Lorestan provinces and OC43 virus in Alborz and Zanjan provinces were detected (data not shown in the figure).

**Table 1.** Age distribution of the children with SARI.

**Age**	**Number**	**WUPyV**	**KIPyV**	**NL63**	**229E**	**HKU1**	**OC43**
<1	122 (59%)	3 (1.5%)	4 (2%)	1 (0.8%)	4 (3.3%)	3 (2.5%)	3 (2.5%)
1–3	47 (23%)	0	0	0	1 (2.1%)	0	0
3–5	37 (18%)	0	0	0	2 (5.4%)	2 (5.4%)	0
Total	206 (100%)	3 (1.5%)	4 (2%)	1 (0.5%)	7 (3.4%)	5 (2.4%)	3 (1.5%)

## DISCUSSION

The current study identified some respiratory viruses in 11.16% of children less than five years of age with SARI who were influenza negative. Coronaviruses were the predominant viruses which their overall prevalence was comparable to the other studies. The second viruses with highest frequency were KIPyV andWUPyV. Parechovirus and HRSV have not been detected in this study.

The findings of this research are consistent with some previous studies. Ligozzia, et al. in Italy reported that of 482 patients aged younger than 5, KIPyV (3.1%) and WUPyV (4.9%) were detected (14). In one study in China, 406 nasopharyngeal aspirate samples were collected from children with acute respiratory symptoms by Yuan et al. The mean age of the patients was about 2 years old, 60% were male and 40% were female. In their study, 2.7% (6 males and 5 females) and 4.2% (9 males and 8 females) of cases were positive for KIPyV and WUPyV, respectively (15). Bialasiewicz et al. in a report of 951 respiratory samples from children under 5 years of age with RTI showed that 24 children (2.5%) were positive for KIPyV (16). In another study in southern China conducted by Zhuang et al. of 771 children with RTI, 15 (2%) were WUPyV positive, with a mean age of 2 to 48 months, 9 of whom were male and 6 were female. Also 4 out of 15 cases had co-infection with HRSV and one case had co-infection with ADV and RV (1). Abed et al. in 157 nasopharyngeal aspirates samples showed that from symptomatic respiratory infected children, 2 (2.5%) out of 79 collected samples were WUPyV positive with mean age of 13 months. Also, 5 out of 78 samples (6.4%) collected from asymptomatic cases, were WUPyV positive with mean age of 20-months (17). A study on respiratory samples by Hormozdi in the United States was conducted which most of them were children under 2 years of age and KIPyV was 2.8% (n = 72), of which 71% (n = 51) had co-infection with viruses such as IFV A and B, PIV, RV, ADV and HRSV. Mean age was 12 months and 68% were male (18).

In a study in Iran in 2012, NL63 was detected only in a 28 days old girl out of 322 respiratory samples (19). In a similar study in China, 0.9% NL63 was detected in 878 respiratory samples from children with ARTIs (20). In another study in China, 4.3% (489/11399) of hospitalized children with ARTIs were positive for HCoV, of which 3.0% were positive for OC43, 0.6% for 229E, 0.5% for NL63, and 0.3% for HKU1 (21). Between, December 2009 and June 2010, in Rural Coastal Kenya, HCoV-NL63 was detected in 1.3% (75/5573) of children with pneumonia (22). In a study of 664 specimens from 592 hospitalized children under six years of age, HCoVs were detected in 6% of samples. Of these specimens, HKU1, OC43, 229E, NL63 were identified in 52.5%, 17.5%, 15% and 15% respectively (23).

In disagreement with present study, Iaria et al. in Italy showed that in three-month follow up of 94 cystic fibrosis patients in different age ranges approximately 337 nasopharyngeal samples were collected. Of these, 12.1% were positive for KIPyV and 8.9% positive for WUPyV (24). Csoma et al. by collecting 532 respiratory swabs in Hungary showed that KIPyV & WUPyV were detected in 14.3% and 9.1% of respiratory samples respectively (10). In one study in Singapore, Hansen-Estruch et al. indicated that among 201 nasopharyngeal aspirate samples of 45-month-old children with respiratory tract infection, WUPyV was positive in 13% (26/201) and KIPyV in 3% (6/201), of which 1% (2/201) was positive for co-infection (9). Rao et al. in Philippines showed that among 1077 nasal samples of patients aged 6 weeks to 5 years with advanced disease of the lower respiratory tract infection, the prevalence and rate of co-infection were 5.3% and 74% for WUPyV and 4.2% and 84% for KIPyV, respectively. The KIPyV viral load was significantly higher in children with severe respiratory disease, but no clear correlation was found between WUPyV viral load and clinical symptoms (25). SX et al. in an epidemiological study on WUPyV and KIPyV among Chinese children with ARI showed that among 3730 samples of nasopharyngeal secretion, 12.14% KIPyV (453/3730) and 1.69% WUPyV (63/3730) were found. The rate of positive cases of co-infection with other respiratory viruses was 2.31% (n = 86) which the highest rate of positive cases was among children under 3 years of age (26). It was shown by Teramoto et al. that in 232 samples of children with RTI, WUPyV and KIPyV were identified in 7 (3%) and 38 (16.4% children, respectively. Age range for positive cases of KIPyV was 3 months to 2 years and for WUPyV was 1 month to 4 years (27).

Parechoviruses were first isolated in 1956 from rectal swabs of infants (28). However, the results of HPeV detection was negative in this study, there are evidence of the virus in gastrointestinal and respiratory illnesses (29). Despite these observations, there is a paucity of data regarding the role of this virus in the respiratory infections. In one study by Sharp et al. in respiratory samples of hospitalized children at Texas State Hospital, HPeV prevalence among 720 nasopharyngeal aspirate samples collected from 637 hospitalized children were 3% (20 children). Fifteen out of 20 samples were HPeV3 and 2 out of 20 were HPeV1. Also, 19 positive cases were under 3 years (30). In another study in the Netherlands, Harvala et al. demonstrated that in 3844 respiratory samples with age range from 6 months to 5 years, 83% were nasopharyngeal aspirates collected from 2200 children, of whom 1211 were male and 989 were female. In their study, in addition to detection of HPeV, common viruses causing RTIs such as: ADV, HRSV, etc. were investigated. In most cases (37%), HPeV co-infected with ADV. Then positive cases were screened to identify HPeV genotypes. Of these, 25 were identified as HPeV1 (HPeV1) and 8 as HPeV6, both of them mostly occur in July and August (29).

In contrast to our study, HRSV is a common respiratory pathogen in children. In a study in Nigeria, 17.2% of children with RTIs were positive for HRSV (31). In a similar study in Philippines, of 1505 children with severe pneumonia, 28.1% had HRSV (6). In a study in China, from 813 children less than 14 years old with ARTIs, HRSV in 40.71%, HCoVHKU1 in 2.21% and HCoV-NL63 in 3.81% were detected which HRSV was the most prevalent respiratory virus (32). In a research by Kwofie et al. HRSV was detected in 14.1% of 128 children under five years of age hospitalized with ARTI (33).

In conclusion the prevalence of studied viruses in this research was somehow similar to the other studies but unexpectedly, in this study HRSV was not detected which is really important among viral respiratory infections in children. As we know RNA viruses are really sensitive to inactivation due to environmental situations and their genome may be degraded (34) then for future studies, we need to use fresh samples for HRSV and HPeV detection.
